# Poly[[penta­aqua­bis­(μ_3_-hydrogen squarato)barium] monohydrate]

**DOI:** 10.1107/S1600536813014736

**Published:** 2013-06-08

**Authors:** Chahrazed Trifa, Amira Bouhali, Sofiane Bouacida, Chaouki Boudaren, Hocine Merazig

**Affiliations:** a Unité de Recherche de Chimie de l’Environnement et Moléculaire Structurale, CHEMS, Université Constantine, 25000 , Algeria; b Département Sciences de la Matière, Faculté des Sciences Exactes et Sciences de la Nature et de la Vie, Université Oum El Bouaghi, Algeria

## Abstract

The crystal structure of the title compound, {[Ba(C_4_HO_4_)_2_(H_2_O)_5_]·H_2_O}_*n*_, consists of discrete double chains propagating along [010]. The chains are formed by Ba^II^ ions linked by bridging hydrogen squarate ligands in a *trans*-bis-monodentate mode. In addition, the bridging hydrogen squarate ligands connect the chains into a ladder structure *via* a third coordinating O atom. The remaining coordination sites are occupied by five aqua ligands and a second mondendate hydrogen squarate ligand, forming a slightly distorted tricapped trigonal–prismatic geometry. O—H⋯O hydrogen bonds link the chains and solvent water mol­ecules into a three-dimensional network.

## Related literature

For the synthesis and applications of cyclic oxocarbons, see: Cohen *et al.* (1959 ▶); Bertolasi *et al.* (2001 ▶). For crystal structures of hydrogen squarate complexes, see: Brach *et al.* (1987 ▶); Uçar *et al.* (2005 ▶); Lee *et al.* (1996 ▶). For related alkaline earth squarates, see: Robl & Weiss (1986*a* ▶,*b* ▶); Koferstein & Robl (2002 ▶). For other related structures, see: Trifa *et al.* (2011 ▶); Bouhali *et al.* (2011 ▶). For the bond-valence method, see: Hormillosa *et al.* (1993 ▶). 
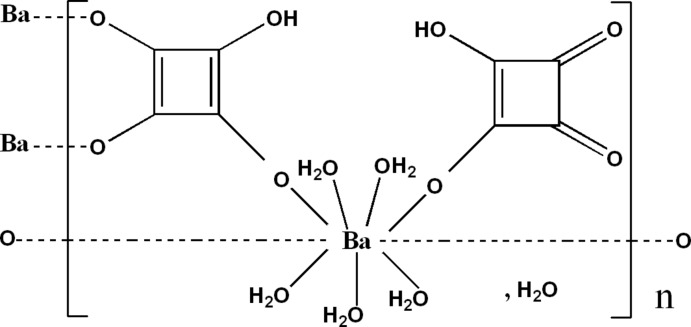


## Experimental

### 

#### Crystal data

[Ba(C_4_HO_4_)_2_(H_2_O)_5_]·H_2_O
*M* *_r_* = 471.53
Monoclinic, 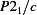

*a* = 11.1522 (11) Å
*b* = 9.0268 (8) Å
*c* = 14.3025 (14) Å
β = 94.009 (5)°
*V* = 1436.3 (2) Å^3^
*Z* = 4
Mo *K*α radiation
μ = 2.84 mm^−1^
*T* = 150 K
0.12 × 0.1 × 0.09 mm

#### Data collection

Bruker APEXII CCD diffractometer
Absorption correction: multi-scan (*SADABS*; Sheldrick, 2002 ▶) *T*_min_ = 0.731, *T*_max_ = 1.000
12082 measured reflections
2550 independent reflections
2471 reflections with *I* > 2σ(*I*)
*R*_int_ = 0.022

#### Refinement

*R*[*F* ^2^ > 2σ(*F* ^2^)] = 0.013
*wR*(*F* ^2^) = 0.034
*S* = 1.06
2550 reflections
264 parameters
All H-atom parameters refined
Δρ_max_ = 0.53 e Å^−3^
Δρ_min_ = −0.25 e Å^−3^

### 

Data collection: *APEX2* (Bruker, 2011 ▶); cell refinement: *SAINT* (Bruker, 2011 ▶); data reduction: *SAINT*; program(s) used to solve structure: *SIR2002* (Burla *et al.*, 2005 ▶); program(s) used to refine structure: *SHELXL97* (Sheldrick, 2008 ▶); molecular graphics: *ORTEP-3 for Windows* (Farrugia, 2012 ▶) and *DIAMOND* (Brandenburg & Berndt, 2001 ▶); software used to prepare material for publication: *WinGX* (Farrugia, 2012 ▶).

## Supplementary Material

Crystal structure: contains datablock(s) global, I. DOI: 10.1107/S1600536813014736/lh5617sup1.cif

Structure factors: contains datablock(s) I. DOI: 10.1107/S1600536813014736/lh5617Isup2.hkl

Additional supplementary materials:  crystallographic information; 3D view; checkCIF report

## Figures and Tables

**Table 1:** Selected bond lengths (Å)

Ba1—O1	2.6857 (10)
Ba1—O3*W*	2.7032 (12)
Ba1—O5*W*	2.7358 (11)
Ba1—O1*W*	2.7500 (12)
Ba1—O2*W*	2.7791 (12)
Ba1—O6	2.7851 (11)
Ba1—O4*W*	2.8356 (14)
Ba1—O3^i^	2.7983 (10)
Ba1—O4^ii^	2.9630 (11)

Symmetry codes: (i) 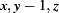; (ii) 
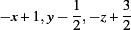
.

**Table 2:** Hydrogen-bond geometry (Å, °)

*D*—H⋯*A*	*D*—H	H⋯*A*	*D*⋯*A*	*D*—H⋯*A*
O1*W*—H1*A*⋯O4*W* ^iii^	0.74 (3)	2.13 (2)	2.8448 (19)	162 (3)
O1*W*—H1*B*⋯O8^iv^	0.86 (3)	1.93 (3)	2.7854 (16)	175 (2)
O2*W*—H2*A*⋯O11*W* ^v^	0.78 (3)	2.22 (3)	2.9431 (17)	156 (2)
O2*W*—H2*B*⋯O7^vi^	0.86 (3)	1.98 (3)	2.8451 (17)	178 (3)
O3*W*—H3*A*⋯O4^i^	0.85 (3)	1.94 (3)	2.7724 (16)	165 (2)
O3*W*—H3*B*⋯O11*W* ^vii^	0.79 (3)	2.05 (2)	2.8160 (17)	165 (2)
O4*W*—H4*A*⋯O7^viii^	0.77 (3)	2.07 (3)	2.7916 (16)	156 (2)
O4*W*—H4*B*⋯O5^ix^	0.80 (2)	2.37 (3)	3.1245 (17)	156 (2)
O5*W*—H5*A*⋯O11*W*	0.84 (3)	1.96 (3)	2.7941 (16)	177 (3)
O5*W*—H5*B*⋯O1^ii^	0.85 (2)	1.87 (2)	2.7176 (15)	173 (3)
O11*W*—H11*A*⋯O8^iv^	0.75 (2)	1.93 (2)	2.6730 (17)	169 (2)
O11*W*—H11*B*⋯O5*W* ^x^	0.85 (2)	1.94 (3)	2.7711 (16)	165 (2)
O2—H21⋯O6	0.86 (3)	1.77 (3)	2.6207 (15)	178 (2)
O5—H51⋯O3^i^	0.87 (2)	1.71 (2)	2.5795 (15)	176 (3)

Symmetry codes: (i) 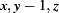; (ii) 
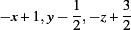
; (iii) 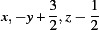; (iv) 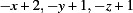; (v) 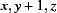; (vi) 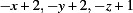; (vii) 
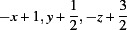
; (viii) 
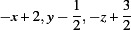
; (ix) 
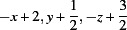
; (x) 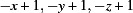.

**Fig. 1.**
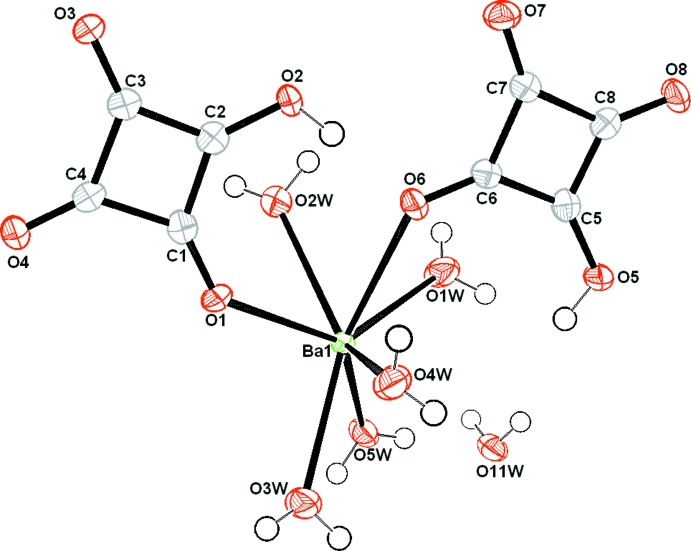
An ORTEP-3 (Farrugia, 2012) drawing of the asymmetric unit (I), with displacement ellipsoids drawn at the 50% probability level.

**Fig. 2.**
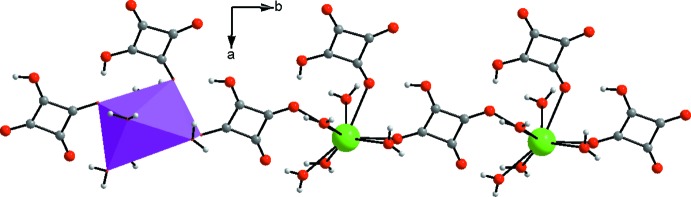
A View of a single chain in (I) along [010].

**Fig. 3.**
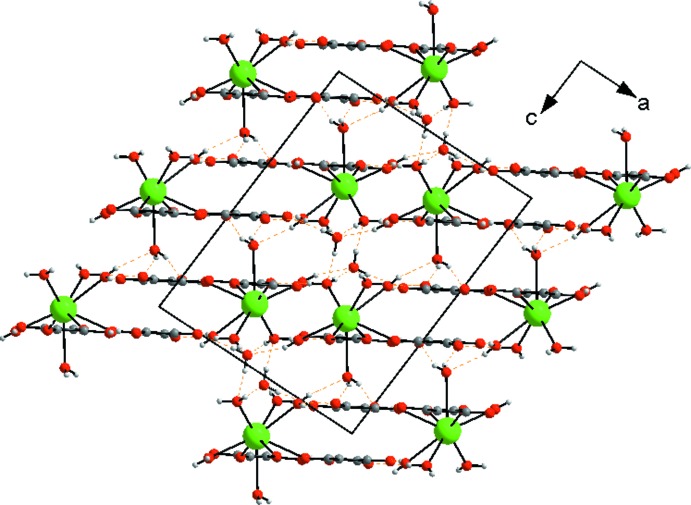
Projection of the structure along the b axis. Dashed lines denote hydrogen bonds.

## supplementary crystallographic information

### Comment

Squaric acid, H_2_C_4_O_4_ (3,4-dihydroxycyclobut-3-ene-1,2-dione, Sq), was synthesized for the first time by Cohen *et al.* in 1959 and has attracted interest because of its cyclic structure and possible aromaticity. It belongs to the series of cyclic oxocarbons of formula H_2_C_n_O_n_ (n = 3–6 for deltic, squaric, croconic and rhodizonic acids, respectively). It and its anions (Hsq- and sq^2-^) are also a useful tools for constructing crystalline architectures and they possess proton donating and accepting capabilities for hydrogen bonding (Cohen *et al.*1959; Bertolasi *et al.* 2001). The molecule presents high degree of electron delocalization, which is very important in crystal packing (Brach *et al.* 1987; Ucar *et al.* 2005; Lee *et al.* 1996). The crystal structure of alkaline earth squarates (Robl *et al.* 1986*a*,*b*; Koferstein & Robl. 2002) have already been published. Recently, we reported the crystal structures of a hemihydrate barium strontium hydrogen squarate (Trifa *et al.* 2011) and strontium hydrogen squarate (Bouhali *et al.* 2011). This paper describes the synthesis and crystal structure of the title compound, (I).

The asymmetric unit of (I) consists of one Ba^II^ ion, two hydrogen squarate anions, five coordinated water molecules and one solvent water molecule (Fig. 1). Each Ba^II^ ion displays a slightly-distorted tricapped trigonal prismatic geometry, defined by four O atoms of two hydrogen squarate anions and five water molecules. The mean value deduced from the Ba–O bonding interactions taken in the range 2.6857 (10)–2.9630 (11) Å agrees with that calculated from the program VALENCE (Hormillosa *et al.* 1993). The C—O bond lengths indicate that the degree of delocalization in the HSQ– ion in (I) is comparable with literature values (Bertolasi *et al.* 2001). The structure of (I) consists of infinite linear chains with composition [Ba(HC_4_O_4_)_2_(H_2_O)_5_]_n_ running along [010] (Fig. 2). The bridging squarate groups adopt two coordination modes, µ-1monodentate and µ-2 *trans* bis monodentate. In the crystal, O—H···O hydrogen bonds link the one-dimenaional chains and solvent water molecules into a three-dimensional network (Fig. 3).

It is particularly interesting to compare the crystal structure of this compound with that of its corresponding hemi hydrate barium strontium hydrogen squarate, [Ba_0.35_Sr_0.65_(HC_4_O_4_)_2_(H_2_O)_5_], 0.5H_2_O (Trifa *et al.* 2011). Indeed both structures can be described by chains connected by hydrogen squarate group, However, we can note the following important differences: the presence of Ba/SrO_9_ polyhedra in the barium strontium hydrogen squarate and a different space group (*C*2/*c*) and lattice parameters. Moreover, due to the higher symmetry, the structure is built from dimers of edge-sharing monocapped square antiprisms [(Ba/Sr)O_3_(H_2_O)_6_].

### Experimental

All chemicals were purchased from commercial sources and used as received without further purification. The title compound, was synthesized by using a hydrothermal method. Typically a mixture of BaCl_2_.2H_2_O (0.112 g) and H_2_C_4_O_4_ (0.114 g) were suspended in H_2_O (*ca* 9 ml). The mixture was then placed in a Teflon lined autoclave, sealed and heated to 393K for 4 days followed by cooling in a water bath. The yellow crystals suitable for X-ray diffraction were filtered, washed with water and dried in air.

### Refinement

All H atoms were located in difference Fourier maps and refined isotropically.

### Crystal data

**Table:**

[Ba(C_4_HO_4_)_2_(H_2_O)_5_]·H_2_O	*F*(000) = 920
*M**_r_* = 471.53	*D*_x_ = 2.181 Mg m^−^^3^
Monoclinic, *P*2_1_/*c*	Mo *K*α radiation, λ = 0.71073 Å
Hall symbol: -P 2ybc	Cell parameters from 9365 reflections
*a* = 11.1522 (11) Å	θ = 2.3–25.1°
*b* = 9.0268 (8) Å	µ = 2.84 mm^−^^1^
*c* = 14.3025 (14) Å	*T* = 150 K
β = 94.009 (5)°	Bloc, yellow
*V* = 1436.3 (2) Å^3^	0.12 × 0.1 × 0.09 mm
*Z* = 4	

### Data collection

**Table:**

Bruker APEXII CCD diffractometer	2550 independent reflections
Radiation source: sealed tube	2471 reflections with *I* > 2σ(*I*)
Graphite monochromator	*R*_int_ = 0.022
φ and ω scans	θ_max_ = 25.1°, θ_min_ = 2.7°
Absorption correction: multi-scan (*SADABS*; Sheldrick, 2002)	*h* = −13→13
*T*_min_ = 0.731, *T*_max_ = 1.000	*k* = −10→10
12082 measured reflections	*l* = −17→17

### Refinement

**Table:**

Refinement on *F*^2^	Primary atom site location: structure-invariant direct methods
Least-squares matrix: full	Secondary atom site location: difference Fourier map
*R*[*F*^2^ > 2σ(*F*^2^)] = 0.013	Hydrogen site location: inferred from neighbouring sites
*wR*(*F*^2^) = 0.034	All H-atom parameters refined
*S* = 1.06	*w* = 1/[σ^2^(*F*_o_^2^) + (0.0174*P*)^2^ + 0.8037*P*] where *P* = (*F*_o_^2^ + 2*F*_c_^2^)/3
2550 reflections	(Δ/σ)_max_ = 0.003
264 parameters	Δρ_max_ = 0.53 e Å^−^^3^
0 restraints	Δρ_min_ = −0.25 e Å^−^^3^

### Special details

**Table:**

Geometry. All e.s.d.'s (except the e.s.d. in the dihedral angle between two l.s. planes) are estimated using the full covariance matrix. The cell e.s.d.'s are taken into account individually in the estimation of e.s.d.'s in distances, angles and torsion angles; correlations between e.s.d.'s in cell parameters are only used when they are defined by crystal symmetry. An approximate (isotropic) treatment of cell e.s.d.'s is used for estimating e.s.d.'s involving l.s. planes.
Refinement. Refinement of *F*^2^ against ALL reflections. The weighted *R*-factor *wR* and goodness of fit *S* are based on *F*^2^, conventional *R*-factors *R* are based on *F*, with *F* set to zero for negative *F*^2^. The threshold expression of *F*^2^ > σ(*F*^2^) is used only for calculating *R*-factors(gt) *etc*. and is not relevant to the choice of reflections for refinement. *R*-factors based on *F*^2^ are statistically about twice as large as those based on *F*, and *R*- factors based on ALL data will be even larger.

### Fractional atomic coordinates and isotropic or equivalent isotropic displacement parameters (Å^2^)

**Table:**

	*x*	*y*	*z*	*U*_iso_*/*U*_eq_	
Ba1	0.676848 (7)	0.760410 (9)	0.685367 (6)	0.00657 (5)	
O4W	0.83608 (12)	0.75740 (13)	0.84885 (10)	0.0117 (3)	
O1	0.67679 (9)	1.02235 (11)	0.77441 (7)	0.0100 (2)	
C3	0.76489 (13)	1.36306 (16)	0.72323 (10)	0.0076 (3)	
O6	0.90411 (9)	0.87276 (11)	0.65721 (7)	0.0098 (2)	
C4	0.66220 (13)	1.30175 (17)	0.77342 (10)	0.0075 (3)	
C1	0.70789 (13)	1.14962 (16)	0.75478 (10)	0.0077 (3)	
O3W	0.56965 (11)	0.65706 (14)	0.83609 (8)	0.0139 (2)	
O2	0.89727 (9)	1.16255 (12)	0.66655 (8)	0.0103 (2)	
O5	0.99133 (10)	0.52766 (11)	0.61707 (8)	0.0114 (2)	
C2	0.80471 (14)	1.21456 (17)	0.70706 (11)	0.0080 (3)	
O2W	0.65618 (12)	0.97776 (13)	0.54757 (8)	0.0154 (2)	
O7	1.13514 (9)	0.98991 (11)	0.55422 (7)	0.0107 (2)	
O1W	0.73539 (11)	0.64714 (14)	0.51598 (9)	0.0155 (3)	
O8	1.20517 (9)	0.64982 (11)	0.50651 (8)	0.0112 (2)	
C7	1.09085 (13)	0.86799 (16)	0.56650 (10)	0.0075 (3)	
C5	1.02281 (13)	0.66277 (17)	0.59606 (11)	0.0083 (3)	
C6	0.98586 (13)	0.81173 (17)	0.61497 (10)	0.0074 (3)	
O3	0.79995 (9)	1.49094 (11)	0.70579 (7)	0.0093 (2)	
C8	1.12363 (14)	0.71099 (17)	0.54615 (11)	0.0083 (3)	
O4	0.57822 (9)	1.35215 (11)	0.81426 (7)	0.0103 (2)	
O11W	0.56817 (12)	0.28422 (13)	0.52709 (8)	0.0116 (2)	
O5W	0.51886 (10)	0.54413 (12)	0.62332 (8)	0.0111 (2)	
H4A	0.860 (2)	0.681 (3)	0.8658 (15)	0.022 (6)*	
H11A	0.634 (2)	0.293 (2)	0.5207 (14)	0.016 (5)*	
H1B	0.7564 (19)	0.556 (3)	0.5121 (15)	0.030 (6)*	
H1A	0.750 (2)	0.685 (3)	0.4720 (18)	0.033 (7)*	
H4B	0.892 (2)	0.813 (3)	0.8462 (16)	0.034 (7)*	
H3A	0.5667 (19)	0.563 (3)	0.8392 (15)	0.031 (6)*	
H5A	0.535 (2)	0.465 (3)	0.5962 (17)	0.040 (7)*	
H5B	0.458 (2)	0.529 (3)	0.6545 (16)	0.037 (6)*	
H3B	0.542 (2)	0.690 (3)	0.8807 (18)	0.035 (7)*	
H11B	0.535 (2)	0.323 (3)	0.4773 (17)	0.032 (6)*	
H2A	0.616 (2)	1.046 (3)	0.5350 (17)	0.043 (7)*	
H2B	0.720 (3)	0.985 (3)	0.5169 (18)	0.045 (7)*	
H51	0.925 (2)	0.518 (3)	0.6447 (18)	0.049 (7)*	
H21	0.901 (2)	1.068 (3)	0.6630 (17)	0.049 (7)*	

### Atomic displacement parameters (Å^2^)

**Table:**

	*U*^11^	*U*^22^	*U*^33^	*U*^12^	*U*^13^	*U*^23^
Ba1	0.00723 (7)	0.00479 (6)	0.00797 (7)	−0.00014 (3)	0.00246 (4)	−0.00034 (3)
O4W	0.0108 (6)	0.0094 (6)	0.0149 (7)	−0.0005 (5)	0.0006 (5)	0.0027 (4)
O1	0.0121 (5)	0.0052 (5)	0.0133 (6)	−0.0007 (4)	0.0048 (4)	0.0005 (4)
C3	0.0077 (7)	0.0086 (7)	0.0062 (7)	−0.0005 (6)	−0.0015 (6)	0.0002 (5)
O6	0.0091 (5)	0.0085 (5)	0.0124 (5)	0.0008 (4)	0.0046 (4)	−0.0009 (4)
C4	0.0082 (7)	0.0072 (7)	0.0068 (7)	−0.0008 (6)	−0.0015 (6)	0.0003 (6)
C1	0.0076 (7)	0.0096 (8)	0.0059 (7)	0.0010 (6)	−0.0008 (6)	−0.0007 (6)
O3W	0.0198 (6)	0.0093 (6)	0.0136 (6)	−0.0004 (5)	0.0085 (5)	−0.0006 (5)
O2	0.0086 (5)	0.0075 (5)	0.0155 (6)	0.0014 (4)	0.0060 (4)	−0.0002 (4)
O5	0.0095 (6)	0.0067 (5)	0.0187 (6)	−0.0007 (4)	0.0062 (5)	0.0012 (4)
C2	0.0077 (8)	0.0085 (7)	0.0074 (8)	−0.0004 (6)	−0.0014 (6)	−0.0004 (6)
O2W	0.0157 (6)	0.0129 (6)	0.0186 (6)	0.0038 (5)	0.0078 (5)	0.0049 (5)
O7	0.0114 (5)	0.0084 (5)	0.0126 (6)	−0.0025 (4)	0.0031 (4)	−0.0007 (4)
O1W	0.0251 (7)	0.0096 (6)	0.0128 (6)	0.0018 (5)	0.0088 (5)	0.0011 (5)
O8	0.0091 (5)	0.0096 (5)	0.0154 (6)	0.0011 (4)	0.0053 (4)	−0.0019 (4)
C7	0.0070 (7)	0.0097 (7)	0.0057 (7)	−0.0003 (6)	−0.0011 (6)	0.0002 (6)
C5	0.0071 (7)	0.0093 (8)	0.0082 (7)	0.0004 (6)	−0.0010 (6)	−0.0010 (6)
C6	0.0059 (7)	0.0094 (7)	0.0067 (7)	−0.0007 (6)	−0.0013 (6)	0.0006 (6)
O3	0.0101 (5)	0.0058 (5)	0.0124 (5)	−0.0004 (4)	0.0033 (4)	0.0003 (4)
C8	0.0083 (8)	0.0081 (7)	0.0082 (7)	−0.0004 (6)	−0.0020 (6)	−0.0001 (6)
O4	0.0098 (5)	0.0082 (5)	0.0136 (6)	0.0006 (4)	0.0050 (4)	−0.0010 (4)
O11W	0.0085 (6)	0.0140 (6)	0.0125 (6)	−0.0003 (5)	0.0031 (5)	0.0018 (5)
O5W	0.0113 (6)	0.0105 (6)	0.0121 (6)	−0.0010 (4)	0.0049 (5)	−0.0018 (4)

### Geometric parameters (Å, º)

**Table:**

Ba1—O1	2.6857 (10)	O3W—H3A	0.85 (3)
Ba1—O3W	2.7032 (12)	O3W—H3B	0.79 (3)
Ba1—O5W	2.7358 (11)	O2—C2	1.3058 (19)
Ba1—O1W	2.7500 (12)	O2—H21	0.85 (3)
Ba1—O2W	2.7791 (12)	O5—C5	1.3099 (19)
Ba1—O6	2.7851 (11)	O5—H51	0.86 (3)
Ba1—O4W	2.8356 (14)	O2W—H2A	0.77 (3)
Ba1—O3^i^	2.7983 (10)	O2W—H2B	0.86 (3)
Ba1—O4^ii^	2.9630 (11)	O7—C7	1.2241 (18)
O3—Ba1^iii^	2.7983 (10)	O1W—H1B	0.86 (3)
O4—Ba1^iv^	2.9630 (11)	O1W—H1A	0.75 (3)
O4W—H4A	0.77 (2)	O8—C8	1.2351 (19)
O4W—H4B	0.80 (3)	C7—C6	1.491 (2)
O1—C1	1.2380 (18)	C7—C8	1.497 (2)
C3—O3	1.2496 (18)	C5—C6	1.438 (2)
C3—C2	1.436 (2)	C5—C8	1.441 (2)
C3—C4	1.499 (2)	O11W—H11A	0.75 (2)
O6—C6	1.2557 (19)	O11W—H11B	0.85 (3)
C4—O4	1.2255 (19)	O5W—H5A	0.84 (3)
C4—C1	1.495 (2)	O5W—H5B	0.85 (3)
C1—C2	1.442 (2)		
			
O1—Ba1—O3W	84.89 (4)	O3—C3—C4	134.17 (14)
O1—Ba1—O5W	139.52 (3)	C2—C3—C4	89.30 (12)
O3W—Ba1—O5W	72.64 (4)	C6—O6—Ba1	127.48 (9)
O1—Ba1—O1W	138.85 (4)	O4—C4—C1	135.01 (14)
O3W—Ba1—O1W	136.03 (4)	O4—C4—C3	136.55 (14)
O5W—Ba1—O1W	68.66 (4)	C1—C4—C3	88.42 (11)
O1—Ba1—O2W	73.26 (3)	O1—C1—C2	135.70 (14)
O3W—Ba1—O2W	142.17 (4)	O1—C1—C4	135.04 (14)
O5W—Ba1—O2W	104.72 (4)	C2—C1—C4	89.25 (12)
O1W—Ba1—O2W	69.56 (4)	Ba1—O3W—H3A	114.0 (14)
O1—Ba1—O6	77.19 (3)	Ba1—O3W—H3B	137.3 (18)
O3W—Ba1—O6	134.32 (3)	H3A—O3W—H3B	109 (2)
O5W—Ba1—O6	141.71 (3)	C2—O2—H21	115.5 (17)
O1W—Ba1—O6	74.54 (3)	C5—O5—H51	116.6 (17)
O2W—Ba1—O6	70.83 (3)	O2—C2—C3	132.07 (14)
O1—Ba1—O3^i^	137.07 (3)	O2—C2—C1	134.86 (14)
O3W—Ba1—O3^i^	81.83 (3)	C3—C2—C1	93.04 (12)
O5W—Ba1—O3^i^	73.36 (3)	Ba1—O2W—H2A	137.9 (18)
O1W—Ba1—O3^i^	67.90 (4)	Ba1—O2W—H2B	112.5 (17)
O2W—Ba1—O3^i^	134.70 (3)	H2A—O2W—H2B	108 (2)
O6—Ba1—O3^i^	83.50 (3)	Ba1—O1W—H1B	119.7 (15)
O1—Ba1—O4W	68.80 (3)	Ba1—O1W—H1A	131 (2)
O3W—Ba1—O4W	68.01 (4)	H1B—O1W—H1A	108 (2)
O5W—Ba1—O4W	127.78 (3)	O7—C7—C6	135.31 (14)
O1W—Ba1—O4W	123.32 (4)	O7—C7—C8	135.78 (14)
O2W—Ba1—O4W	127.42 (4)	C6—C7—C8	88.76 (11)
O6—Ba1—O4W	66.36 (3)	O5—C5—C6	137.96 (14)
O3^i^—Ba1—O4W	68.36 (3)	O5—C5—C8	128.76 (14)
O1—Ba1—O4^ii^	73.80 (3)	C6—C5—C8	93.13 (12)
O3W—Ba1—O4^ii^	67.47 (3)	O6—C6—C5	136.74 (15)
O5W—Ba1—O4^ii^	66.64 (3)	O6—C6—C7	133.99 (14)
O1W—Ba1—O4^ii^	113.11 (3)	C5—C6—C7	89.21 (12)
O2W—Ba1—O4^ii^	76.82 (3)	C3—O3—Ba1^iii^	131.74 (9)
O6—Ba1—O4^ii^	141.44 (3)	O8—C8—C5	135.85 (15)
O3^i^—Ba1—O4^ii^	134.97 (3)	O8—C8—C7	135.24 (14)
O4W—Ba1—O4^ii^	123.17 (3)	C5—C8—C7	88.86 (11)
Ba1—O4W—H4A	116.7 (16)	C4—O4—Ba1^iv^	131.52 (9)
Ba1—O4W—H4B	113.6 (17)	H11A—O11W—H11B	103 (2)
H4A—O4W—H4B	109 (3)	Ba1—O5W—H5A	127.3 (16)
C1—O1—Ba1	134.44 (9)	Ba1—O5W—H5B	117.8 (16)
O3—C3—C2	136.52 (14)	H5A—O5W—H5B	108 (2)
			
O3W—Ba1—O1—C1	−176.77 (14)	O3—C3—C2—C1	178.50 (18)
O5W—Ba1—O1—C1	−121.24 (13)	C4—C3—C2—C1	−0.12 (12)
O1W—Ba1—O1—C1	−1.98 (16)	O1—C1—C2—O2	−0.4 (3)
O2W—Ba1—O1—C1	−28.01 (13)	C4—C1—C2—O2	178.04 (18)
O6—Ba1—O1—C1	45.53 (13)	O1—C1—C2—C3	−178.29 (18)
O3^i^—Ba1—O1—C1	111.03 (13)	C4—C1—C2—C3	0.12 (12)
O4W—Ba1—O1—C1	114.86 (14)	Ba1—O6—C6—C5	−27.9 (2)
O4^ii^—Ba1—O1—C1	−108.77 (14)	Ba1—O6—C6—C7	155.81 (13)
O1—Ba1—O6—C6	178.40 (12)	O5—C5—C6—O6	−3.4 (3)
O3W—Ba1—O6—C6	108.87 (12)	C8—C5—C6—O6	−178.92 (18)
O5W—Ba1—O6—C6	−15.47 (14)	O5—C5—C6—C7	173.96 (19)
O1W—Ba1—O6—C6	−31.82 (11)	C8—C5—C6—C7	−1.56 (11)
O2W—Ba1—O6—C6	−105.11 (12)	O7—C7—C6—O6	3.1 (3)
O3^i^—Ba1—O6—C6	37.00 (12)	C8—C7—C6—O6	178.98 (17)
O4W—Ba1—O6—C6	106.18 (12)	O7—C7—C6—C5	−174.39 (18)
O4^ii^—Ba1—O6—C6	−139.67 (11)	C8—C7—C6—C5	1.50 (11)
O3—C3—C4—O4	−0.1 (3)	C2—C3—O3—Ba1^iii^	155.33 (14)
C2—C3—C4—O4	178.63 (18)	C4—C3—O3—Ba1^iii^	−26.6 (2)
O3—C3—C4—C1	−178.57 (17)	O5—C5—C8—O8	3.0 (3)
C2—C3—C4—C1	0.12 (11)	C6—C5—C8—O8	179.15 (18)
Ba1—O1—C1—C2	−38.1 (3)	O5—C5—C8—C7	−174.60 (16)
Ba1—O1—C1—C4	144.19 (13)	C6—C5—C8—C7	1.55 (11)
O4—C4—C1—O1	−0.2 (3)	O7—C7—C8—O8	−3.3 (3)
C3—C4—C1—O1	178.31 (17)	C6—C7—C8—O8	−179.12 (18)
O4—C4—C1—C2	−178.67 (18)	O7—C7—C8—C5	174.36 (18)
C3—C4—C1—C2	−0.12 (11)	C6—C7—C8—C5	−1.50 (11)
O3—C3—C2—O2	0.5 (3)	C1—C4—O4—Ba1^iv^	−43.2 (2)
C4—C3—C2—O2	−178.13 (17)	C3—C4—O4—Ba1^iv^	138.94 (15)

Symmetry codes: (i) *x*, *y*−1, *z*; (ii) −*x*+1, *y*−1/2, −*z*+3/2; (iii) *x*, *y*+1, *z*; (iv) −*x*+1, *y*+1/2, −*z*+3/2.

### Hydrogen-bond geometry (Å, º)

**Table:**

*D*—H···*A*	*D*—H	H···*A*	*D*···*A*	*D*—H···*A*
O1*W*—H1*A*···O4*W*^v^	0.74 (3)	2.13 (2)	2.8448 (19)	162 (3)
O1*W*—H1*B*···O8^vi^	0.86 (3)	1.93 (3)	2.7854 (16)	175 (2)
O2*W*—H2*A*···O11*W*^iii^	0.78 (3)	2.22 (3)	2.9431 (17)	156 (2)
O2*W*—H2*B*···O7^vii^	0.86 (3)	1.98 (3)	2.8451 (17)	178 (3)
O3*W*—H3*A*···O4^i^	0.85 (3)	1.94 (3)	2.7724 (16)	165 (2)
O3*W*—H3*B*···O11*W*^iv^	0.79 (3)	2.05 (2)	2.8160 (17)	165 (2)
O4*W*—H4*A*···O7^viii^	0.77 (3)	2.07 (3)	2.7916 (16)	156 (2)
O4*W*—H4*B*···O5^ix^	0.80 (2)	2.37 (3)	3.1245 (17)	156 (2)
O5*W*—H5*A*···O11*W*	0.84 (3)	1.96 (3)	2.7941 (16)	177 (3)
O5*W*—H5*B*···O1^ii^	0.85 (2)	1.87 (2)	2.7176 (15)	173 (3)
O11*W*—H11*A*···O8^vi^	0.75 (2)	1.93 (2)	2.6730 (17)	169 (2)
O11*W*—H11*B*···O5*W*^x^	0.85 (2)	1.94 (3)	2.7711 (16)	165 (2)
O2—H21···O6	0.86 (3)	1.77 (3)	2.6207 (15)	178 (2)
O5—H51···O3^i^	0.87 (2)	1.71 (2)	2.5795 (15)	176 (3)

Symmetry codes: (i) *x*, *y*−1, *z*; (ii) −*x*+1, *y*−1/2, −*z*+3/2; (iii) *x*, *y*+1, *z*; (iv) −*x*+1, *y*+1/2, −*z*+3/2; (v) *x*, −*y*+3/2, *z*−1/2; (vi) −*x*+2, −*y*+1, −*z*+1; (vii) −*x*+2, −*y*+2, −*z*+1; (viii) −*x*+2, *y*−1/2, −*z*+3/2; (ix) −*x*+2, *y*+1/2, −*z*+3/2; (x) −*x*+1, −*y*+1, −*z*+1.
